# On the notion of false advertising: A reply to the letter by Theodore C. Chan and Gary M. Vilke on our article “Studying asphyxiation in the lab: the role of experimental evidence in cause-of-death inquiry”

**DOI:** 10.1007/s40656-026-00762-1

**Published:** 2026-09-24

**Authors:** Enno Fischer, Saana Jukola

**Affiliations:** 1 Dresden University of Technology, Zellescher Weg 17, 01069 Dresden, Germany; 2 University of Twente, Drienerlolaan 5, 7522 Enschede, The Netherlands

**Keywords:** Evidence, Experiment, False advertising, Forensic medicine, Asphyxiation, Excited Delirium Syndrome

## Abstract

Theodore Chan and Gary Vilke criticize our arguments concerning a series of lab-based studies on asphyxiation. In this reply to their letter, we explain that Chan and Vilke’s letter misrepresents our arguments in several important ways. We also clarify the notion of false advertising that we employed in our initial analysis and explain why some of the studies regarding asphyxiation represent instances of this problematic practice.

## Dear Editors

We read with interest the letter to the editors by Theodore Chan and Gary Vilke (Chan & Vilke, 2026) and appreciate this opportunity to respond to their criticisms of our article “Studying asphyxiation in the lab: the role of experimental evidence in cause-of-death inquiry” (Fischer & Jukola, 2026).

To begin with, the letter misrepresents some of our claims in the article. First, Chan and Vilke write “[…] one wonders how these authors can […] pass comparative judgement, casting such aspersions and accusations of ‘false claims’ and ‘intended misrepresentations’[.]” Nowhere do we even use the term ‘false claims’ that the letter quotes. Neither do we argue that the mentioned studies are intentional misrepresentations. In fact, we are careful to point out that what we identify as false advertising can happen without such intentions. Second, the letter quotes us as stating that mechanistic evidence ranks “close to the bottom” in the evidence hierarchy. We would like to clarify that with this we reported a common view expressed in the literature on Evidence Based Medicine (EBM), a view that we have argued faces severe limitations in the current context of forensic medicine.

We agree with Chan and Vilke that our discussion of Reay et al.’s (1988) initial study on asphyxiation can be usefully contextualized by referencing Reay’s later work from 1998. But it should be noted that Chan and Vilke’s letter cites the article by Reay very unfavorably, if not misleadingly. It is true that Reay concedes that the 1988 study was “refuted” in “normal individuals”. However, in the next sentence of the 1998 article (not quoted in the letter by Chan and Vilke) Reay goes on to restrict this claim, pointing out that additional issues arise, in particular, for individuals with an abundant abdominal panniculus or scoliosis (Reay 1998, p. 14). We think it is exactly this practice of making selective claims and citations that leads to the issues of false advertising that we discuss in our article.

In our article we were referring to Martin Carrier’s (2013) use of the term “false advertising” that describes a mismatch between the goal of a study and its methodology. More specifically, it describes instances in which the “procedures used are not floppy or inappropriate in general [...] The procedures are highly suitable for revealing false positives, that is, they are good at ruling out the mistaken assumption that health risks exist. However, the question they purport to answer is the converse one, namely, to ascertain that false negatives are avoided. The result suggested or intimated is that no health risks exist” (p. 2560).

Accordingly, we did not state in our article that the methods used by Chan et al. (2004) are problematic as such. More specifically, we did not criticize Chan et al.’s study as an attempt of showing that the Reay et al. (1988) study was a false positive. What we do criticize, however, is that the specific claims that the study advances are sometimes taken to support much broader claims about whether certain restraint positions can be excluded as causing or even contributing to death. Importantly, these broader claims have been put forward also by some of the authors of Chan et al. (2004).^1^

Here it is essential to not just consider individual claims about the impact of restraint positions in oxygenation and ventilation under lab conditions but to also observe the wider context in which research on asphyxiation stands, as Chan and Vilke rightly point out. Part of this wider context is an ongoing debate about the “Excited Delirium Syndrome” (ExDS). While some have argued that ExDS may explain certain instances of death-in-custody, others have rejected such claims and argued that ExDS lacks scientific validity and may mask deaths that are due to excessive police violence (for details see, e.g., Fischer & Jukola (2024)). In this context, the study by Chan et al. (2004) and similar studies have frequently been invoked to exclude asphyxiation as a cause of death and to support the competing hypothesis that death was caused by ExDS. For example, Charles Wetli argues that “there are now numerous studies that indicate the methods of police restraint, with or without pepper spray or pressure on the back, have nothing to do with the death (2006).^2^ In a similar vein, DiMaio and DiMaio (2006) argue that the “concept that most deaths due to excited delirium syndrome are due to positional or restraint asphyxia was dealt what many consider a death blow in 1997 by the work of Chan et al.” (p. 23, the cited study is Chan et al. (1997)). It is this wider context, in which, we believe, the false advertising of claims made by asphyxiation studies needs to be prevented.

Sincerely,

Enno Fischer and Saana Jukola

## Data Availability

Not applicable.

## Footnotes

1 Here are two examples: In an article from 2007 it is argued that “factors other than ventilatory failure associated with the restraining process may be responsible for the sudden unexpected deaths of restrained individuals” (Michalewicz et al. 2007, p. 174). In an article from 2013 it is argued that “another cause of cardiovascular collapse is more likely in these cases than decreased CO [cardiac output] secondary to prone position with weight force” (Savaser et al. 2013, p. 994).

2 Wetli is citing Chan et al. (2004) among other studies. His paper appeared in a volume co-edited by Chan.
